# Increased Resilience Weakens the Relationship between Perceived Stress and Anxiety on Sleep Quality: A Moderated Mediation Analysis of Higher Education Students from 7 Countries

**DOI:** 10.3390/clockssleep2030025

**Published:** 2020-08-11

**Authors:** Chen Du, Megan Chong Hueh Zan, Min Jung Cho, Jenifer I. Fenton, Pao Ying Hsiao, Richard Hsiao, Laura Keaver, Chang-Chi Lai, HeeSoon Lee, Mary-Jon Ludy, Wan Shen, Winnie Chee Siew Swee, Jyothi Thrivikraman, Kuo-Wei Tseng, Wei-Chin Tseng, Robin M. Tucker

**Affiliations:** 1 Department of Food Science and Human Nutrition, Michigan State University, East Lansing, MI 48824, USA; duchen@msu.edu (C.D.); imigjeni@msu.edu (J.I.F.); 2 Division of Nutrition and Dietetics, International Medical University, Kuala Lumpur 57000, Malaysia; megan_chong@imu.edu.my (M.C.H.Z.); winnie_chee@imu.edu.my (W.C.S.S.); 3 Global Public Health, Leiden University College, 2595 DG The Hague, The Netherlands; m.j.cho@luc.leidenuniv.nl (M.J.C.); j.k.thrivikraman@luc.leidenuniv.nl (J.T.); 4 Department of Food and Nutrition, Indiana University of Pennsylvania, Indiana, PA 15705, USA; pyhsiao@iup.edu; 5 Department of Kinesiology, Health, and Sport Science, Indiana University of Pennsylvania, Indiana, PA 15705, USA; hsiao@iup.edu; 6 Department of Health and Nutritional Science, Institute of Technology Sligo, F91 YW50 Sligo, Ireland; keaver.laura@itsligo.ie; 7 Department of Exercise and Health Sciences, University of Taipei, Taipei, Taiwan; sports_injury0406@yahoo.com.tw (C.-C.L.); fossil0405@yahoo.com.tw (K.-W.T.); speedceng@gmail.com (W.-C.T.); 8 Department of Human Services, Bowling Green State University, Bowling Green, OH 43403, USA; leeh@bgsu.edu; 9 Department of Public & Allied Health, Bowling Green State University, Bowling Green, OH 43403, USA; mludy@bgsu.edu (M.-J.L.); wanshen@bgsu.edu (W.S.)

**Keywords:** COVID-19, sleep, stress, anxiety, rumination, psychological resilience, university students

## Abstract

High levels of perceived stress and anxiety among university students are a global concern and are known to negatively influence sleep. However, few studies have explored how stress response styles, like psychological resilience and rumination, might alter these relationships. Using validated tools, perceived stress, anxiety, stress response styles, and sleep behaviors of undergraduate and graduate students from seven countries during the height of the COVID-19 pandemic were characterized in order to examine the relationships between these factors using mediation and moderation analyses. Students enrolled in universities in China, Ireland, Malaysia, Taiwan, South Korea, the Netherlands, and the United States were recruited in May 2020. A total of 2254 students completed this cross-sectional study. Perceived stress and anxiety were negatively associated with sleep quality through the mediation of rumination. Increased psychological resilience weakened the relationships between perceived stress and anxiety on sleep quality. The majority of students reported that COVID-19 negatively influenced their mental health and sleep quality but not sleep duration. Based on these results, university students would likely benefit from sleep education and mental health promotion programs that include trainings to increase psychological resilience and reduce rumination, particularly during times of increased stress.

## 1. Introduction

Worldwide, university students report experiencing high levels of perceived stress and anxiety [1,2,3,4,5]. Perceived stress reflects an individual’s perception of how stressful their life is currently, regardless of how objectively stressful it might be [6]. Individuals perceiving elevated levels of stress over a sustained period are at risk for numerous chronic physical and mental illnesses, including cardiovascular disease, hypertension, depression, and anxiety disorders [7,8,9,10,11]. Given the lengthy time course for the development of most chronic diseases and the (typically) young age of the student population, mental health disorders present a more immediate concern. Anxiety and anxiety disorders are particularly prevalent among undergraduate and graduate student populations, frequently surpassing depression [3,8,9]. For example, in Malaysia, 45% of undergraduate students surveyed were classified as suffering from moderate or severe anxiety while 14% were moderately or severely depressed [2]. Across four years of undergraduate education, Chinese students suffered more from anxiety than depression in one recent longitudinal study [3]. Other studies reported that one-quarter of Taiwanese students identified as being anxious [4], and nearly 40% of Irish students perceived their anxiety to be severe [12]. Further, among American graduate students, 41% reported moderate or severe anxiety [5]. Based on these examples, high levels of perceived stress and anxiety are clearly global phenomena among students in higher education.

Elevated levels of perceived stress and anxiety can lead to insufficient and poor-quality sleep [13,14,15]. Insufficient and poor-quality sleep are independent risk factors for a multitude of chronic diseases [16,17] and may provide a mechanism by which stress and anxiety negatively impact health outcomes. Negative associations between sleep duration and quality and perceived stress have been well documented [18,19,20,21,22], and a temporal relationship between perceived stress and sleep is suggested in the literature [13,15,23]. That is, increased stress precedes sleep problems. This relationship is supported by intervention studies that demonstrated poorer sleep quality after increasing participants’ stress [15,23], while reducing stress improved sleep quality [23]. Stress appears to decrease restorative sleep phases, including slow wave sleep and rapid eye movement sleep; decrease sleep efficiency; and increase awakenings [15]. Similar to perceived stress, the relationship between sleep quality and duration and anxiety has long been studied [13,24,25,26], and as with stress, heightened anxiety typically precedes difficulty sleeping [13]. Taken together, these studies support the notion that higher levels of perceived stress and anxiety frequently result in insufficient or poor-quality sleep.

Rumination is a maladaptive response style to a stressful situation, whereby, an individual tends to repeatedly think about or fixate on the situation [27]. Previous studies demonstrated that a higher tendency toward rumination was associated with insomnia and poor sleep quality [28,29,30,31]. Recent work also demonstrated that rumination could serve as a potential mediator between stressful life events and sleep problems [32,33], and post-stressor rumination predicted longer sleep-onset latency [34]. Therefore, the degree of rumination could affect the relationships between stress, anxiety, and sleep.

Unlike rumination, higher levels of psychological resilience (referred to as resilience from here on) characterized by the ability to quickly recover from a stressful event, are associated with the ability to better manage stress [35,36]. Several studies suggested that resilience can reduce the adverse effects of stress on mental health and sleep [37,38,39,40]. Individuals with higher levels of resilience tended to cope better with adversity, and resilience served as a protective factor against negative psychological outcomes such as stress, depression, and anxiety [37,38,39,40,41,42]. Thus, varying levels of psychological resilience likely change how stress and anxiety influence sleep.

When complex relationships like those between perceived stress, anxiety, sleep, rumination, and resilience need to be explored, mediation and moderation analyses are useful statistical tools. Mediation analysis models a relational sequence of one independent variable to a mediating variable (mediator) and then the mediating variable to the dependent (outcome) variable [43]. Moderation analysis examines the strength of the relationship between two variables under different values of a moderating variable (moderator) [44]. Given the complex relationships between stress, anxiety, stress response styles, and sleep behaviors [13,14,15,28,29,30,31,37,38,39,40,41,42], multiple mediation and moderation analysis models were proposed to examine relationships between perceived stress and anxiety on sleep under the mediation of rumination and the moderation of resilience.

The current study’s aim was to explore the relationships between mental health indices (perceived stress and anxiety), sleep, and stress response styles among undergraduate and graduate students in seven countries: China, Ireland, Malaysia, South Korea, Taiwan, the Netherlands, and the United States. Consequently, the objectives of the study were to (1) examine whether rumination mediated the relationships between perceived stress and sleep quality or duration, (2) examine whether rumination mediated the relationships between anxiety and sleep quality or duration, (3) examine whether resilience moderated the direct and the indirect relationships between perceived stress and sleep quality or duration, and (4) examine whether resilience moderated the direct and the indirect relationships between anxiety and sleep quality or duration. Data collection occurred during the height of the COVID-19 pandemic: between late April and May 31, 2020. This time period was selected as popular reports suggested (e.g., [45]), and the researchers hypothesized, this was a particularly stressful time for students due to academic, family, financial, and occupational concerns, such that, the investigated mental health indices were likely to be negatively impacted. Based on the documented relationships between stress, anxiety, rumination, resilience, and sleep, hypotheses included:

(1) Higher levels of perceived stress would be associated with decreased sleep quality and duration through increased rumination.
(2) Higher levels of anxiety would be associated with decreased sleep quality and duration through increased rumination.
(3) Students with higher levels of resilience were likely to be protected from the negative impact of perceived stress and anxiety on sleep quality and sleep duration.

**Figure 1:**
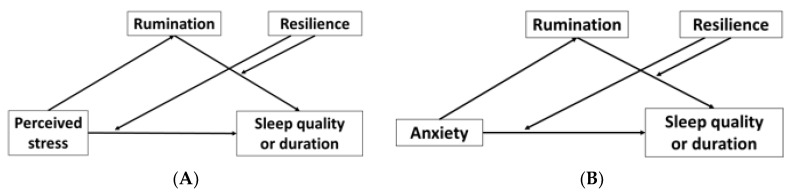
Proposed moderated mediation models. (**A**) Proposed moderated mediation models of perceived stress on sleep quality (Model 1) and perceived stress on sleep duration (Model 2). (**B**) Proposed moderated mediation models of anxiety on sleep quality (Model 3) and anxiety on sleep duration (Model 4).

To test the hypotheses, four moderated mediation models were proposed (Figure 1). The models were built to first test whether rumination mediates the relationship between perceived stress and sleep quality and duration and whether rumination mediates the relationship between anxiety and sleep quality and duration. Secondly, the models sought to examine whether resilience moderates the direct and indirect effects of perceived stress and anxiety on sleep quality and duration. Direct and indirect effects are statistical terms to describe the relationships between two variables, where direct effects describe how the two variables are directly correlated, and indirect effects describes how the two variables are indirectly correlated through a mediator.

## 2. Results

### 2.1. Participant Characteristics

A total of 2254 participants from seven countries completed the survey (Table 1). The majority of the participants were female (66.7%), undergraduate students (79.9%), and were studying in their own countries (87.0%), i.e., not international students. The average age of the participants was 22.5 ± 5.5 years and the average body mass index (BMI) was 24.4 ± 5.6 kg/m^2^. Most participants met the recommended minimum daily sleeping duration of 7 h (72.2%) but were classified as poor sleepers (60.3%) based on Pittsburgh Sleep Quality Index (PSQI) scores > 5 (Table 2). Nearly 40% of students (36.4%) reported moderate to severe anxiety, and more than four-fifths (85.0%) of participants reported moderate or high levels of perceived stress (Table 2).

### 2.2. Mental Health and Sleep Behaviors Influenced by COVID-19

In general, COVID-19 negatively influenced university students’ mental health and sleep behaviors. More than half of the students reported greater perceived stress, greater repetitive negative thinking, greater negative mood, and greater anxiety during COVID-19 compared to before COVID-19 (Table 3). In addition, 41.7% of the students reported greater financial stress and 29.5% reported reduced resilience to stress during COVID-19. In terms of sleep behaviors, most of the students slept more or slept the same amount (82.9%) during COVID-19 compared to before the pandemic; however, 32.1% of students reported reduced sleep quality (Table 3). In summary, perceived stress, anxiety, and rumination increased for the majority of the university students, and resilience and sleep quality declined for some university students during COVID-19 compared to before.

### 2.3. Mediation and Moderation Analyses

#### 2.3.1. Correlations between Examined Variables

The Pearson zero-order correlation analyses showed that sleep quality, indicated by PSQI score, was significantly correlated with sleep duration, resilience, rumination, perceived stress, and anxiety (Table 4). The higher the PSQI score, the worse the sleep quality; therefore, lower sleep quality was correlated with lower resilience scores, higher rumination scores, higher perceived stress scores, and higher anxiety scores. The relationships between sleep quality and rumination scores, perceived stress scores, and anxiety scores were moderate (0.4 < |*r*| < 0.7), and the relationship between sleep quality and resilience was weak (|*r*| < 0.4). Lower sleep quality was also significantly but weakly correlated with shorter sleep duration. In addition, sleep duration was negatively but weakly correlated with anxiety. Resilience was negatively and moderately correlated with rumination, perceived stress, and anxiety. Rumination was positively and moderately correlated with perceived stress and anxiety. Perceived stress was positive and moderately correlated with anxiety. Age was negatively but weakly correlated with sleep duration, rumination, perceived stress, and BMI. Age was positively but weakly correlated with resilience. Further, BMI was negatively but weakly correlated with sleep duration and rumination and positively but weakly correlated with sleep quality (PSQI), rumination, perceived stress, and anxiety.

#### 2.3.2. Model 1 Moderated Mediation Model of Perceived Stress on Sleep Quality

The mediation analysis of Model 1 showed a significant direct effect of perceived stress on sleep quality, and perceived stress was significantly and positivity associated with rumination. However, rumination was not significantly associated with sleep quality (Table 5). The significant indirect effect of perceived stress on sleep quality through its effect on rumination indicated that rumination significantly mediated the relationship between perceived stress and sleep quality. Even though rumination was not significantly associated with sleep quality, rumination was still a significant mediator due to the fact that the mediation of rumination does not depend on both the relationship between perceived stress and rumination and the relationship between rumination and sleep quality being significant [46].

The moderation analyses showed that Interaction 1 (perceived stress and resilience) was significantly associated with sleep quality, but resilience alone and Interaction 2 (rumination and resilience) were not associated with sleep quality (Table 6). This indicated that the moderation effect of resilience was only significant for the direct relationship between perceived stress and sleep. As resilience increased, the association between perceived stress and sleep weakened, and when the resilience score was above 4.61, the direct effect of perceived stress on sleep quality was no longer significant according to the Johnson-Neyman test.

The conditional indirect effect test for resilience revealed that at a wide range of resilience scores, from mean minus one standard deviation to mean plus one standard deviation, the relationship between perceived stress and sleep quality, through its effect on rumination, did not change. Similarly, rumination continued to be a significant mediator of perceived stress and sleep quality regardless of the level of resilience. The Johnson-Neyman test was not performed since resilience did not moderate the indirect relationship between perceived stress and sleep quality.

To summarize, the mediation and moderation analyses of Model 1 demonstrated that the greater amount of perceived stress a student experienced, the poorer their sleep quality, and this was associated with increased rumination. However, as psychological resilience increased, the negative relationship between perceived stress and sleep quality weakened and eventually disappeared when the resilience score was higher than 4.61.

#### 2.3.3. Model 2 Moderated Mediation Model of Perceived Stress on Sleep Duration

The mediation analysis of Model 2 showed a non-significant direct effect of perceived stress on sleep duration. Perceived stress was significantly, and positivity associated with rumination, but rumination was not significantly associated with sleep duration (Table 7). The indirect effect of perceived stress on sleep duration through its effect on rumination was also not significant. Therefore, rumination did not mediate the relationship between perceived stress and sleep duration.

The moderation analyses showed that resilience, Interaction 1 (perceived stress and resilience), and Interaction 2 (rumination and resilience) were not associated with sleep duration (Table 8). This indicated that resilience did not moderate the direct or the indirect relationship between perceived stress and sleep duration. Further, the conditional indirect and direct effect tests results confirmed that at a wide range of resilience, from mean minus one standard deviation to mean plus one standard deviation, the relationship between perceived stress to sleep duration and the relationship between perceived stress to sleep duration through its effect on rumination did not change. Due to resilience not being a significant moderator of the effects of perceived stress on sleep duration, the Johnson-Neyman test was not performed.

To summarize, the mediation and moderation analyses of Model 2 demonstrated that the amount of stress that students perceived was not associated with their sleep duration, and the level of psychological resilience did not alter the relationship between perceived stress and sleep duration.

#### 2.3.4. Model 3 Moderated Mediation Model of Perceived Stress on Sleep Quality

The mediation analysis of Model 3 showed a significant direct effect of anxiety on sleep quality, and anxiety was significantly and positivity associated with rumination. However, rumination was not significantly associated with sleep quality (Table 9). The significant indirect effect of anxiety on sleep quality through its effect on rumination indicated that rumination significantly mediated the relationship between perceived stress and sleep quality. Even though rumination was not significantly associated with sleep quality, rumination was still a significant mediator due to the fact that the mediation of rumination does not depend on both the relationship between anxiety and rumination and the relationship between rumination and sleep quality being significant [46].

The moderation analyses showed that resilience, Interaction 1 (anxiety and resilience), and Interaction 2 (rumination and resilience) were not significantly associated with sleep quality (Table 10). The conditional indirect effect test of resilience revealed that as resilience scores increased, the significant indirect effect of anxiety on sleep quality through its effect on rumination did not change. Further, the conditional direct effect of resilience showed that as resilience scores increased, the direct effect of anxiety on sleep quality declined but remained significant. Therefore, the Johnson-Neyman test was performed to test the conditional direct effect of resilience. The test results confirmed that as resilience scores increased, the strength of the relationship between anxiety and sleep quality, as measured by PSQI, decreased. However, there was no statistically significant transition point within the possible ranges of resilience scores, which indicated that higher resilience scores weakened the negative relationship between anxiety and sleep quality (shown as a positive correlation between anxiety and poor sleep quality due to sleep quality being measured using PSQI), but increased resilience score did not make the significant relationship disappear at any level of resilience.

To summarize, the mediation and moderation analyses of Model 3 demonstrated that the more anxiety students experienced, the poorer their sleep quality was, and this was associated with increased rumination. High psychological resilience could serve as a buffer to reduce the negative relationship between anxiety and sleep quality, but high levels of resilience alone did not diminish the relationship.

#### 2.3.5. Model 4 Moderated Mediation Model of Perceived Stress on Sleep Quality

The mediation analysis of Model 4 showed that there was no significant direct effect of anxiety on sleep duration. Anxiety was significantly and positively associated with rumination, but rumination was not significantly associated with sleep duration (Table 11). The indirect effect of anxiety on sleep duration through its effect on rumination was also not significant. Therefore, rumination did not mediate the relationship between anxiety and sleep duration.

The moderation analyses showed that resilience, Interaction 1 (anxiety and resilience), and Interaction 2 (rumination and resilience) were not associated with sleep duration (Table 12). This indicated that resilience did not moderate the direct and indirect relationships between anxiety and sleep duration. Further, the conditional indirect effect test confirmed that at a wide range of resilience scores, from mean minus one standard deviation to mean plus one standard deviation, the relationship between anxiety and sleep duration through its effect on rumination did not change and were all non-significant. The conditional direct effect of the resilience test showed that the direct negative effect of anxiety on sleep duration was significant when resilience score was at mean minus one standard deviation (2.46) and at the mean value (3.18), but nonsignificant when resilience score was at mean plus one standard deviation (3.9). Even though there was a change in statistical significance of the direct relationship between anxiety and sleep duration as resilience score changes, resilience did not moderate the direct effect of anxiety on sleep duration due to the nonsignificant direct effect of anxiety on sleep. The Johnson-Neyman test was not performed since the direct effect of anxiety on sleep duration was not significant.

To summarize, the mediation and moderation analyses of Model 4 demonstrated that the amount of anxiety that students experienced was not associated with sleep duration, and the resilience score did not alter the relationship between anxiety and sleep duration.

## 3. Discussion

This study sought to characterize relationships between perceived stress, anxiety, rumination, resilience, and sleep. The analyses demonstrated that perceived stress and anxiety were negatively associated with sleep quality, but not sleep duration, and these relationships were mediated by rumination. Psychological resilience appears to serve as a buffer to weaken the negative relationships between perceived stress and anxiety on sleep quality. Therefore, training to decrease rumination and improve resilience among university students would appear to improve sleep quality; however, this still needs to be empirically tested.

As hypothesized, COVID-19 negatively impacted university students’ perceived stress and anxiety. These findings are similar to previous reports that large-scale disasters including infectious pandemics were accompanied by increases in post-traumatic stress disorder (PTSD), depression, anxiety, apprehension, substance abuse, and other mental and behavioral disorders [47,48,49,50]. In addition, one recent study investigated the mental health status among college students in China during the COVID-19 epidemic and noted that 0.9% of the participants were experiencing severe anxiety, 2.7% moderate anxiety, and 21.3% mild anxiety during the epidemic [51]. In the present study, 16.2% of the students reported experiencing severe anxiety, 20.0% moderate anxiety, and 32.0% mild anxiety. While both studies used the same anxiety tool, the differences in anxiety levels could be attributed to the different study populations and timing of the survey. The present study was conducted well into the pandemic, and more information about the spread and mortality of COVID-19 could have induced more stress and anxiety in our population. Mental health issues among university students were already prevalent before COVID-19 [3,5], and the pandemic appears to have exacerbated these problems based on the majority of participants in the current study reporting greater stress and anxiety during the study period.

Worldwide, the student population is at high risk for insufficient sleep and problems with sleep quality, which raises concerns about their overall health. One large American study observed 36% of students did not meet sleep recommendations [52], and among students seeking treatment at campus mental health clinics in the US, nearly 16% indicated that sleep was a concern [53]. In addition, the mean PSQI scores for a group of 300 undergraduate and graduate students from South Korea was 6.5 ± 3.0, which was well over the cut-off score of 5 or less indicating good sleep [54]. The majority (60.3%) of students in the present study were classified as poor sleepers, which is consistent with what has been reported in the literature [55,56,57], and the average PSQI score of the students was 6.8 ± 3.5. In addition, 27.8% of the students failed to meet the minimum recommended sleep duration of 7 h of sleep per day even though 44.6% of the students reported sleeping more during COVID-19 compared to before. The percentage of students who did not meet sleep duration recommendations in the current study is slightly higher than the reported percentage from a study conducted in the Netherlands, where 21.5% of students failed to sleep at least 7 h/night [58]. Although both insufficient sleep duration and poor sleep quality are concerns among university students, poor sleep quality was more widespread.

Both short sleep duration and poor sleep quality increase risks of poor health outcomes such as obesity, mental illness, cardiovascular diseases (CVD), type 2 diabetes, and cancer [56,59,60,61,62,63,64]. While much emphasis has been placed on getting enough sleep, sleep quality might actually be more important than sleep duration in terms of influencing health outcomes [65,66,67]. Meta-analyses conducted among young and older adults reported poor sleep quality, compared to short sleep duration, led to greater odds of being obese and greater risk of developing diabetes, anxiety, and depression [63,65,67]. These studies combined with findings from the current study suggest that in order to improve health outcomes, addressing only sleep duration is not sufficient; sleep quality also needs to be targeted and is possibly more important than sleep duration.

Rumination is known to mediate the relationship between mental health and sleep quality [39,68]. Studies of young adults and college students reported rumination mediated the relationships between depressed mood and stressful life events on sleep quality [39,68]. These studies agree with the findings of the present study in that increased rumination could explain why perceived stress, anxiety, and sleep quality were negatively correlated among university students. However, the present study observed that perceived stress and anxiety were not associated with sleep duration, and rumination did not mediate the relationships between perceived stress or anxiety on sleep duration. Sleep duration in the present study accounted for both weekday and weekend sleep duration, and previous work reported that university students sleep significantly more on weekends compared to weekdays [69], so our duration totals might be skewed higher. Future work should explore the effects of weekday versus weekend sleep and social jet lag on relationships between perceived stress and anxiety, and sleep duration. Therefore, decreasing rumination through mental health promotion or counseling interventions [70,71] might improve university students’ sleep quality, but not sleep duration.

The current study suggests that improving the psychological resilience of university students could also reduce the negative relationships of perceived stress and anxiety on sleep quality but not sleep duration. The higher the resilience, the weaker the relationships between perceived stress or anxiety and sleep quality. Previous studies that utilized multiple regression and mediation and moderation analyses reported similar results to the present study, that is, resilience could buffer the interaction between perceived stress and sleep disturbance among adult and student populations [39,72]. In addition, investigations of mechanisms along with interventional studies suggested that perceived stress and anxiety lead to poor sleep quality [13,15,23]. Based on the temporal relationships between stress and anxiety and sleep, along with the current findings that resilience might buffer the relationships, university students will likely benefit from resilience training as a coping strategy to reduce the negative effects of stress and anxiety on sleep quality.

Despite the effects that insufficient sleep and poor sleep quality have on physical and mental health, sleep problems receive little attention from health professionals. One American study of campus counseling centers that tracked over 161,000 students and 1.2 million appointments observed that less than 3% of the mental health practitioners surveyed reported prioritizing sleep as the primary issue among students who complained of sleep-related problems [53,73]. Nearly 16% of these clients indicated that sleep was a concern [53]. Poor sleep habits predicted poor academic performance to an equal or greater degree than stress or substance abuse in one study [73]; yet, stress management and substance abuse treatment receive far more attention than sleep as universities typically provide counseling services for these specific issues but not for sleep [73]. Improving sleep among students is a pressing concern, and it is one that should be addressed.

Calling for improving sleep among students is useful only if sleep can, in fact, be improved. Based on the results of one recent meta-analysis, sleep education programming has been shown to be effective in improving sleep outcomes among students [74]. Programs were more likely to be effective when based on cognitive behavioral therapy [74]. For universities without sufficient resources to provide cognitive behavioral therapy for insomnia (CBTi), online delivery of CBTi has been shown to be effective according to a recent systematic review [75]. Further, one Dutch study suggested that group-delivered CBTi was also effective [71]. Thus, effective, feasible options for improving sleep outcomes among students are available and, therefore, should be utilized more frequently.

While sleep education programs have been shown to be effective, as stated above, we argue that these programs should also incorporate training designed to improve resilience and decrease rumination. Based on previous work, providing students with education and tools, particularly meditation and mindfulness skills, to promote these outcomes is achievable [76,77,78]. A classroom-based intervention designed to improve coping skills and cognitive responses to stress reduced stress while improving coping and attitudes among American students [76]. Students in the U.K. who received mental health care combined with mindfulness training reported lower distress compared to students who received mental health care alone [77]. These data suggest that teaching stress management skills can improve the quality of life for many students. It is likely that combining this training with sleep education would increase the effectiveness of sleep education programming.

In terms of study strengths, the large sample size allowed for adequate power to conduct the analyses. Surveys were collected from seven countries, which increases the generalizability of the results. Mediation and moderation analyses allowed for a detailed examination of the complex relationships between stress, anxiety, stress response styles, and sleep. Finally, all instruments utilized in the study to measure mental health indices and sleep behaviors were well validated in many countries [6,79,80,81,82].

There are limitations to the study. First, the study was cross-sectional; therefore, findings suggest relational rather than causal sequences between the variables examined. A longitudinal investigation following the current study procedure is recommended in the future to better infer the causal relationships among mental health indices, resilience, rumination, and sleep. Second, the questions examining how COVID-19 influenced mental health and sleep behaviors were not validated. COVID-19 is an emerging pandemic; therefore, validated questionnaires addressing the specific questions examined in this study were not available. Third, weight and height information were self-reported in the study due to pandemic-related prohibition of in-person testing. Fourth, it is possible that for some individuals, the relationship between perceived stress or anxiety and sleep could be reversed, that is, poor sleep outcomes could induce stress and anxiety. However, the majority of the literature suggests that perceived stress and anxiety precede poor sleep, which provides an evidence-based foundation for the models used. Fifth, the study did not control for confinement due to differences in social restrictions across and within countries, although the survey was conducted at the same time in all locations. To fully understand the findings reported here, future work detailing these relationships under post-COVID-19 conditions are recommended. Finally, surveys were administered in English, so students required language proficiency to participate.

## 4. Materials and Methods

### 4.1. Study Design

University students enrolled in universities in China, Ireland, Malaysia, Taiwan, South Korea, the Netherlands, and the United States were recruited into this cross-sectional study. The online survey was administered in April and May 2020, during the COVID-19 pandemic. At this time, most states in the United States were under shelter in place orders [83]. Ireland, Malaysia, and the Netherlands had also enacted shelter in place orders in most areas. China, Taiwan, and South Korea had just lifted the shelter in place orders, so some personnel and students at the universities had returned to work and school (Table 1).

Eligible participants were university students, including undergraduate, graduate/professional, domestic, and international students, who were at least 18 years old. The study was approved by Michigan State University Human Research Protection Program (East Lansing, MI, USA), International Medical University Joint Committee on Research and Ethics (Kuala Lumpur, Malaysia), Faculty of Governance and Global Affairs Ethics Committee (The Hague, South Holland, Netherlands), Indiana University Institutional Review Board for the Protection of Human Subjects (Indiana, PA, USA), Institute Research Ethics Committee, Institute of Technology, Sligo (Sligo, Ireland), Institutional Review Board of University of Taipei (Taipei, Taiwan), and Bowling Green State University Office of Research Compliance (Bowling Green, OH, USA).

### 4.2. Demographics

Demographic information regarding age, gender, major or field of study, university year classification, and domestic vs. international status was collected. Self-reported weight and height were also collected.

### 4.3. Assessment of Perceived Stress and Anxiety

Perceived stress was assessed using the validated Perceived Stress Scale-10 (PSS-10), which provides a global measure of perceived stress during the past month [6]. Anxiety was assessed using the validated Generalized Anxiety Disorder Screener (GAD-7) [79]. The GAD-7 asks about anxiety symptoms over the past two weeks.

### 4.4. Assessment of Psychological Resilience and Rumination

Psychological resilience was assessed using the Brief Resilience Scale (BRS) [84], and rumination was evaluated using the Repetitive Negative Thinking Questionnaire [81]. Both tools are validated for use with the general population [80,84].

### 4.5. Assessment of Sleep Duration and Quality

Assessment of sleep was performed using the Pittsburgh Sleep Quality Index (PSQI), which is a validated tool that measures sleep quality and patterns over the past month [81]. Habitual sleep duration, which did not distinguish between weekday or weekend sleep, was extracted from the PSQI.

### 4.6. Assessment of How COVID-19 Impacted the Factors Described Above

At the end of each survey section, a question about how COVID-19 had impacted the participant’s perceived stress, anxiety, sleep quality, and sleep duration was asked.

### 4.7. Statistical Analysis

Data were analyzed using IBM SPSS Version 26 (IBM Corporation, Armonk, NY, USA). Only completed surveys were included in data analysis. Sample size calculation for mediation and moderation analysis was based on 20 samples per construct (variable) tested in each model [46,85]. A minimum of 80 participants were needed for each model. Descriptive statistics were performed, and data are presented as means ± standard deviation (SD). Correlations were examined between perceived stress, anxiety, rumination, psychological resilience, sleep duration, sleep quality, age, gender, and BMI. Bonferroni correction was performed to determine the adjusted p value to detect significance for multiple comparisons. A total of 28 correlation tests were performed; therefore, the adjusted p value was 0.0018 (0.05 ÷ 28). Moderated mediation analyses were conducted using the SPSS PROCESS Macro developed by Hayes [86,87]. PROCESS was performed for each model by entering one independent variable (perceived stress or anxiety), one mediator (rumination), one moderator (resilience), and one dependent variable (sleep quality or sleep duration). Age, gender, and BMI were entered as covariates for each model. The number of bootstraps performed for bias corrected bootstrap confidence intervals was 10,000. The normality of each variable entered in the model was checked, and all variables were approximately normally distributed after excluding outliers, which was defined by above or below mean±3SD. To conduct the moderated mediation analyses, the following conditions were used: (1) if a mediator significantly mediates the relationship between an independent variable and a dependent variable, the indirect effect of the independent variable on the dependent variable must be significant; (2) if a moderator significantly moderates the relationship between a mediator and a dependent variable, then the effect of the mediator on the dependent variable must be significant; (3) the conditional indirect effect of an independent variable on a dependent variable via a mediator depends on the presence of a certain range of the mediator; and (4) if a moderator significantly moderates the effect between an independent variable and a dependent variable, the effect of the dependent variable on the independent variable must be significant and a statistically significant transition point must be identified using the Johnson-Neyman method, which is used to identify a range of values for a moderator under which that the relationship between an independent variable and a dependent variable is not significant [88]. Statistical significance was determined by *p* < 0.05 for all analyses and 95% confidence interval (CI) not crossing zero for the indirect effect testing of the mediation analyses.

## 5. Conclusions

The current study demonstrated that perceived stress and anxiety were negatively associated with sleep quality, but not sleep duration, through the mediating effects of rumination, and that improving resilience could provide a means to weaken these negative associations. These findings suggest that the incorporation of resilience and rumination management training into sleep education and mental health promotion programs among university students would likely contribute to improved student health. These conclusions need to be tested using interventional approaches. Future studies should focus on examining other mental health indices and other health behaviors, such as dietary habits and physical activity, and should be longitudinal in nature in order to better infer causal relationships.

## Figures and Tables

**Table 1:** Demographic and social restriction information.

Location	Social Restriction Measures in Place	Sex *n* (%)	Undergraduate vs. Graduate *n* (%)	Domestic vs. International *n* (%)
China	Some online classes and some in-person classes resumed	M = 36 (32.4) F = 75 (67.6) Other = 0	U = 94 (84.7) G = 17 (15.3)	D = 106 (95.5) I = 5 (4.5)
Ireland	Online classes only	M = 53 (27.6) F = 138 (71.9) Other = 1 (0.5)	U = 154 (80.2) G = 38 (19.8)	D = 179 (93.2) I = 13 (6.8)
Malaysia	Online classes only	M = 19 (20.9) F = 72 (79.1) Other = 0	U = 88 (96.7) G = 3 (3.3)	D = 74 (81.3) I = 17 (18.7)
South Korea	Some online classes and some in-person classes resumed	M = 54 (60.7) F = 35 (39.3) Other = 0	U = 41(46.1) G = 48 (53.9)	D = 84 (94.4) I = 5 (5.6)
Taiwan	Some online classes and some in-person classes resumed	M = 202 (53.6) F = 155 (41.1) Other = 20 (5.3)	U = 360 (95.5) G = 17 (4.5)	D = 360 (95.5) I = 17 (4.5)
The Netherlands	Online classes only	M = 22 (19.3) F = 92 (80.7) Other = 0	U = 114 (100) G = 0	D = 46 (40.4) I = 68 (59.6)
United States	Online classes only	M = 308 (24.1) F = 935 (73.0) Other = 37 (2.0)	U = 951 (74.3) G = 327 (25.7)	D = 1113 (87.0) I = 167 (13)
**Total**		**M = 694 (30.8)** **F = 1502 (66.7)** **Other = 58 (2.5)** **Total = 2254**	**U = 1802 (79.9)** **G = 452 (20.1)**	**D = 1962 (87.0)** **I = 292 (13.0)**

Note: *n* = number of participants; M = male, F = female; U = undergraduate students, G = graduate students and post-undergraduate/professional school students; D = domestic students, I = international students. Universities surveyed included Hangzhou Normal University in China; Athlone Institute of Technology (AIT), Institute of Technology Sligo (IT Sligo), Letterkenny Institute of Technology (LYIT), Trinity College Dublin (TCD), University of Limerick (UL), Waterford Institute of Technology (WIT), Institute of Technology Tralee (ITT), Dublin City University (DCU), University College Dublin (UCD), Hibernia College, National University of Galway (NUIG), Technological University Dublin (TUD/TU Dublin), Cork Institute of Technology (CIT), Galway-Mayo Institute of Technology (GMIT), University College Cork (UCC), and Griffith College Dublin in Ireland; International Medical University in Malaysia; Hanyang University, Chungnam National University, Seokyeong University, and University of Seoul in South Korea; University of Taipei in Taiwan; Leiden University College in the Netherlands; Michigan State University, Bowling Green State University, and Indiana University of Pennsylvania in the United States.

**Table 2:** Anxiety, perceived stress, sleep quality, and sleep duration of university students.

Health Parameters	Category	*n* (%)
**Anxiety**	Minimal anxiety	711 (31.6)
	Mild anxiety	722 (32.0)
	Moderate anxiety	456 (20.2)
	Severe anxiety	364 (16.2)
**Perceived stress**	Low stress	337 (15.0)
	Moderate stress	1425 (63.2)
	High stress	492 (21.8)
Sleep quality *	Good sleeper	890 (39.7)
	Poor sleeper	1352 (60.3)
**Sleep duration**	Met ≥ 7 h of sleep per day	1628 (72.2)
	Did not meet the above sleep duration recommendation	626 (27.8)

* Note that sleep quality is based on Pittsburgh Sleep Quality Index (PSQI) score where ≤5 is classified as a good sleeper and >5 is classified as a poor sleeper.

**Table 3:** Percentage of university students who experienced worsened self-reported stress, mental health indices, and sleep behaviors during the COVID-19 pandemic.

	*n*	Greater Perceived Stress (%)	Greater Financial Stress (%)	Reduced Resilience to Stress (%)	Greater Repetitive Negative Thinking (%)	Greater Negative Mood (%)	Greater * Anxiety (%)	Reduced Sleep Duration (%)	Increased Sleep Duration (%)	Reduced Sleep Quality (%)
Total	2254	60.2	41.7	29.5	50.1	50.9	63.9	17.1	44.6	32.1
Undergraduate	1802	59.3	41.6	29.9	50.0	50.6	64.3	17.5	44.9	32.2
Graduate	452	63.7	42.0	28.1	50.4	52.2	62.8	15.5	44.0	31.6
Domestic	1962	60.2	40.9	29.3	49.3	51.0	64.0	16.8	44.2	31.3
International	292	59.6	46.9	30.8	55.1	50.0	62.9	19.2	47.3	37.3

* Due to technical difficulties of data collection, anxiety change during COVID-19 was not recorded for University of Taipei students. The total number of students included in anxiety assessment was 1877 with 1442 undergraduate students and 435 graduate students; 1602 domestic students and 275 international students.

**Table 4:** Zero-order correlations between all outcome measures, mediators, moderators, and covariates for the total sample.

Measures ^a^	1	2	3	4	5	6	7	8
(1) Sleep quality (PSQI) ^b^	-	−0.33 *	−0.28 *	0.41 *	0.43 *	−0.50 *	0.04	0.19 *
(2) Sleep duration (hours)		-	0.05	−0.05	−0.05	−0.08 *	−0.11 *	−0.07 *
(3) Resilience			-	−0.47 *	−0.50 *	−0.42 *	0.09 *	−0.27
(4) Rumination				-	0.62 *	0.67 *	−0.09 *	0.09 *
(5) Perceived stress					-	0.69 *	−0.12 *	0.10 *
(6) Anxiety						−	−0.05	0.16 *
(7) Age (years)							-	0.19 *
(8) BMI (Kg/m^2^)								-
Mean ± SD	6.8 ± 3.5	7.5 ± 1.2	3.2 ± 0.7	82.9 ± 23.0	20.6 ± 6.8	8.2 ± 5.8	22.5 ± 5.5	24.4 ± 5.6

^a^ Numbers in parentheses correspond to column numbers; ^b^ Sleep quality is based on PSQI. Higher PSQI scores indicate poorer sleep quality. * Indicates a significant correlation at the *p* < 0.0018 level based on Bonferroni adjustment for multiple comparisons. BMI = body mass index; SD = standard deviation. Note: PSQI ranges from 0 to 21. Good sleeper is defined by having a PSQI score ≤ 5, and poor sleeper is defined by having a PSQI score > 5. Resilience ranges from 0 to 5. Rumination ranges from 27 to 135. Perceived stress ranges from 0 to 40. Low stress is classified as having a perceived stress score from 0 to 13; moderate stress is from 14 to 26; high stress is from 27 to 40. Anxiety ranges from 0 to 21. Minimal anxiety is classified as having an anxiety score from 0 to 4; mild anxiety is from 5 to 9; moderate anxiety is from 10 to 14; severe anxiety is equal or above 15.

**Table 5:** Model 1 mediation analysis.

Variables	B	SE	*t*	*p* Value
Perceived stress → rumination	2.04	0.05	37.90	<0.001
Rumination → sleep quality	0.01	0.02	0.75	0.46
Perceived stress → sleep quality	0.35	0.06	5.91	<0.001
**Bootstrap**	Effect	SE	LL 95% CI	UL 95% CI
Rumination	0.06	0.01	0.05	0.08

Note: B = unstandardized beta; SE = standard error for the unstandardized beta; *t* = *t* test statistics; LL95%CI = lower limit of 95% confidence interval; UL95%CI = upper limit of 95% confidence interval.

**Table 6:** Model 1 moderation analysis.

Variables	B	SE	*t*	*p* Value
Resilience → sleep quality	0.69	0.36	1.89	0.06
Interaction 1 → sleep quality	−0.06	0.02	−3.68	<0.001
Interaction 2 → sleep quality	0.01	0.01	1.03	0.30
**Conditional indirect effect of resilience**	Resilience scores	Indirect effect (SE)	LL 95% CI	UL 95% CI
	2.46	0.19 (0.02)	0.15	0.23
	3.18	0.14 (0.01)	0.12	0.17
	3.90	0.10 (0.02)	0.06	0.13
**Conditional direct effect of resilience Johnson-Neyman test**	Resilience scores	Direct effect (SE)	LL 95% CI	UL 95% CI
	1.00	0.29 (0.04)	0.05	0.08
	1.80	0.23 (0.03)	0.18	0.29
	2.60	0.18 (0.02)	0.15	0.22
	3.40	0.13 (0.01)	0.10	0.16
	4.20	0.08 (0.02)	0.04	0.12
	4.40	0.07 (0.02)	0.02	0.11
	4.61	0.05 (0.03)	0.00	0.10
	4.80	0.04 (0.03)	−0.02	0.10
	5.00	0.03 (0.03)	−0.04	0.09

Note: B = unstandardized beta; SE = standard error for the unstandardized beta; *t* = *t* test statistics; LL95%CI = lower limit of 95% confidence interval; UL95%CI = upper limit of 95% confidence interval. Interaction 1: perceived stress and resilience; Interaction 2: rumination and resilience.

**Table 7:** Model 2 mediation analysis.

Variables	B	SE	*t*	*p* Value
Perceived stress → rumination	2.04	0.05	37.90	<0.001
Rumination → sleep duration	0.01	0.01	1.14	0.25
Perceived stress → sleep duration	−0.03	0.02	−1.08	0.28
**Bootstrap**	Effect	SE	LL 95% CI	UL 95% CI
Rumination	−0.002	0.003	−0.01	0.001

Note: B = unstandardized beta; SE = standard error for the unstandardized beta; *t* = *t* test statistics; LL95%CI = lower limit of 95% confidence interval; UL95%CI = upper limit of 95% confidence interval.

**Table 8:** Model 2 moderation analysis.

Variables	B	SE	*t*	*p* Value
Resilience → sleep duration	0.21	0.16	1.34	0.18
Interaction 1 → sleep duration	0.01	0.01	0.83	0.40
Interaction 2 → sleep duration	−0.003	0.002	−1.38	0.17
**Conditional indirect effect of resilience**	Resilience scores	Indirect effect (SE)	LL 95% CI	UL 95% CI
	2.46	0.002 (0.005)	−0.008	0.012
	3.18	−0.002 (0.003)	−0.008	0.004
	3.90	−0.007 (0.004)	−0.015	0.001
**Conditional direct effect of resilience**	Resilience scores	Direct effect (SE)	LL 95% CI	UL 95% CI
	2.46	−0.011 (0.008)	−0.027	0.004
	3.18	−0.007 (0.005)	−0.017	0.003
	3.90	−0.003 (0.007)	−0.016	0.011

Note: B = unstandardized beta; SE = standard error for the unstandardized beta; *t* = *t* test statistics; LL95%CI = lower limit of 95% confidence interval; UL95%CI = upper limit of 95% confidence interval. Interaction 1: perceived stress and resilience; Interaction 2: rumination and resilience.

**Table 9:** Model 3 mediation analysis.

Variables	B	SE	*t*	*p* Value
Anxiety → rumination	2.60	0.06	41.23	<0.001
Rumination → sleep quality	0.01	0.02	0.78	0.43
Anxiety → sleep quality	0.35	0.07	5.03	<0.001
**Bootstrap**	Effect	SE	LL 95% CI	UL 95% CI
Rumination	0.05	0.01	0.03	0.07

Note: B = unstandardized beta; SE = standard error for the unstandardized beta; *t* = *t* test statistics; LL95%CI = lower limit of 95% confidence interval; UL95%CI = upper limit of 95% confidence interval.

**Table 10:** Model 3 moderation analysis.

Variables	B	SE	*t*	*p* Value
Resilience → sleep quality	−0.13	0.36	−0.36	0.72
Interaction 1 → sleep quality	−0.04	0.02	−1.88	0.06
Interaction 2 → sleep quality	0.002	0.005	−1.88	0.73
**Conditional indirect effect of resilience**	Resilience scores	Indirect effect (SE)	LL 95% CI	UL 95% CI
	2.46	0.05 (0.01)	0.02	0.07
	3.18	0.05 (0.01)	0.03	0.07
	3.90	0.05 (0.01)	0.03	0.08
**Conditional direct effect of resilience**	Resilience scores	Direct effect (SE)	LL 95% CI	UL 95% CI
	2.46	0.25 (0.02)	0.21	0.29
	3.18	0.22 (0.02)	0.19	0.25
	3.90	0.19 (0.02)	0.15	0.24
**Conditional direct effect of resilience Johnson-Neyman test**	Resilience scores	Direct effect (SE)	LL 95% CI	UL 95% CI
	1.00	0.31 (0.05)	0.21	0.40
	1.80	0.28 (0.03)	0.21	0.34
	2.60	0.24 (0.02)	0.21	0.28
	3.40	0.21 (0.02)	0.18	0.24
	4.20	0.18 (0.03)	0.14	0.23
	4.40	0.17 (0.03)	0.11	0.23
	4.60	0.16 (0.03)	0.10	0.23
	5.00	0.15 (0.04)	0.06	0.23

Note: B = unstandardized beta; SE = standard error for the unstandardized beta; *t* = *t* test statistics; LL95%CI = lower limit of 95% confidence interval; UL95%CI = upper limit of 95% confidence interval. Interaction 1: perceived stress and resilience; Interaction 2: rumination and resilience.

**Table 11:** Model 4 mediation analysis.

Variables	B	SE	*t*	*p* Value
Anxiety → rumination	2.61	0.06	41.24	<0.001
Rumination → sleep duration	0.01	0.01	1.00	0.32
Anxiety → sleep duration	−0.02	0.03	−0.75	0.45
**Bootstrap**	Effect	SE	LL 95% CI	UL 95% CI
Rumination	0.001	0.004	−0.007	0.009

Note: B = unstandardized beta; SE = standard error for the unstandardized beta; *t* = *t* test statistics; LL95%CI = lower limit of 95% confidence interval; UL95%CI = upper limit of 95% confidence interval.

**Table 12:** Model 4 moderation analysis.

Variables	B	SE	*t*	*p* Value
Resilience → sleep duration	0.26	0.17	1.55	0.12
Interaction 1 → sleep duration	0.002	0.010	0.19	0.85
Interaction 2 → sleep duration	−0.002	0.003	−1.00	0.32
**Conditional indirect effect of resilience**	Resilience scores	Indirect effect (SE)	LL 95% CI	UL 95% CI
	2.46	0.006 (0.007)	−0.007	0.019
	3.18	0.001 (0.004)	−0.007	0.009
	3.90	−0.004 (0.006)	−0.015	0.008
**Conditional direct effect of resilience**	Resilience scores	Direct effect (SE)	LL 95% CI	UL 95% CI
	2.46	−0.019 (0.009)	−0.036	−0.001
	3.18	−0.017 (0.006)	−0.029	−0.005
	3.90	−0.016 (0.010)	−0.035	0.003

Note: B = unstandardized beta; SE = standard error for the unstandardized beta; *t* = *t* test statistics; LL95%CI = lower limit of 95% confidence interval; UL95%CI = upper limit of 95% confidence interval. Interaction 1: perceived stress and resilience; Interaction 2: rumination and resilience.
